# Overexpression of CDC25B, CDC25C and phospho-CDC25C (Ser216) in vulvar squamous cell carcinomas are associated with malignant features and aggressive cancer phenotypes

**DOI:** 10.1186/1471-2407-10-233

**Published:** 2010-05-25

**Authors:** Zhihui Wang, Claes G Trope, Vivi Ann Flørenes, Zhenhe Suo, Jahn M Nesland, Ruth Holm

**Affiliations:** 1 Department of Pathology, Oslo University Hospital and University of Oslo, Norway; 2 Department of Oncology, The First Affiliated Hospital of Zhengzhou University, Medical College of Zhengzhou University, Zhengzhou, China; 3 Department of Obstetrics and Gynecology, Oslo University Hospital and University of Oslo, Norway; 4 Department of Pathology, Oslo University Hospital, Norway

## Abstract

**Background:**

CDC25 phosphatases are important regulators of the cell cycle. Their abnormal expression detected in a number of tumors implies that their dysregulation is involved in malignant transformation. However, the role of CDC25s in vulvar cancer is still unknown. To shed light on their roles in the pathogenesis and to clarify their prognostic values, expression of CDC25A, CDC25B and CDC25C in a large series of vulvar squamous cell carcinomas were examined.

**Methods:**

Expression of CDC25A, CDC25B, CDC25C and phosphorylated (phospho)-CDC25C (Ser216) were examined in 300 vulvar carcinomas using immunohistochemistry. Western blot analysis was utilized to demonstrate CDC25s expression in vulvar cancer cell lines. Kinase and phosphatase assays were performed to exclude cross reactivity among CDC25s isoform antibodies.

**Results:**

High nuclear CDC25A and CDC25B expression were observed in 51% and 16% of the vulvar carcinomas, respectively, whereas high cytoplasmic CDC25C expression was seen in 63% of the cases. In cytoplasm, nucleus and cytoplasm/nucleus high phospho-CDC25C (Ser216) expression was identified in 50%, 70% and 77% of the carcinomas, respectively. High expression of CDC25s correlated significantly with malignant features, including poor differentiation and infiltration of vessel for CDC25B, high FIGO stage, presence of lymph node metastases, large tumor diameter, poor differentiation for CDC25C and high FIGO stage, large tumor diameter, deep invasion and poor differentiation for phospho-CDC25C (Ser216). In univariate analysis, high expression of phospho-CDC25C (Ser216) was correlated with poor disease-specific survival (p = 0.04). However, such an association was annulled in multivariate analysis.

**Conclusions:**

Our results suggest that CDC25C and phospho-CDC25C (Ser216) play a crucial role and CDC25B a minor role in the pathogenesis and/or progression of vulvar carcinomas. CDC25B, CDC25C and phospho-CDC25C (Ser216) were associated with malignant features and aggressive cancer phenotypes. However, the CDC25s isoforms were not independently correlated to prognosis.

## Background

Vulvar carcinoma, counting for 3-5% of all female genital cancers [1], is a disease most frequently observed in elder women. However, recently an increase in its incidence was also seen among younger women [2,3]. Although surgery is still kept as the standard treatment [3], considerable morbidity is often inevitably raised as a consequence of radical surgery [4]. In a bid to decrease the incidence of complications, there has been a movement towards individualized therapy and less radical surgery. In this sense, knowledge of biomolecular markers would be of considerable value to yield a better treatment decision.

CDC25 phosphatases, which are believed to be important regulators of cell cycle progression, dominate entry into mitosis by regulating the activation of CDK1/cyclin B [5]. Catalyzed by these dual specificity phosphatases, cyclin/CDKs are dephosphorylated and actived after removal of inhibitory phosphate groups from Thr^14 ^and Tyr^15 ^[6]. In human, three isoforms of CDC25 denoted CDC25A, CDC25B and CDC25C exist. Initially, CDC25A is found to act at the G1/S transition, whereas CDC25B and CDC25C mainly play their roles at the G2/M transition [5,7]. However, recent studies suggest that all three CDC25 phosphatases function as regulators of both G1/S and G2/M transitions [7].

Although exact reasons of tumorigenesis remain unknown, it is believed that one of the hallmarks of tumorigenesis is dysregulation of cell proliferation, and thus is strongly suggested to be connected with disorders of cell cycle [6,8-10]. CDC25s are implied to be involved in the malignant transformation when deficient checkpoints are performed during mitosis [6,11]. The activity of the CDC25s are regulated by their phosphorylation status, expression level and subcellular localization [6,11]. Previously, abnormal expression of CDC25s have been reported in a number of carcinomas, such as breast [12], ovarian [13], esophageal [14], prostate [15] and colorectal carcinomas [9]. Overexpression of CDC25 isoforms are supposed to contribute to tumorigenesis by enhancing tumor malignancy [5]. To our knowledge, expression of CDC25s in vulvar cancers has not yet been reported. The aims of our study were to determine expression statuses of CDC25A, CDC25B and CDC25C in a large series of vulvar squamous cell carcinomas to shed light on their roles in the pathogenesis of this cancer type and to clarify their potential prognostic values.

## Methods

### Patient materials

A retrospective study including 300 cases of vulvar squamous cell carcinoma was performed. These patients underwent resection at The Norwegian Radium Hospital from 1977 to 2006. The median age at diagnosis was 74 years (range 35-96 years). Pre-surgery treatment was given to 9 patients, of which 6 received radiotherapy, whereas the other 3 were treated with radiotherapy/chemotherapy. Two hundred and one (67%) patients received radical vulvectomy. Postoperative treatment including irradiation, chemotherapy and irradiation/chemotherapy were performed on 63 (21%), 3 (1%), and 4 (1%) of the patients, respectively. Relapse was observed in 107 (36%) patients. All patients were followed up since confirmed diagnosis until death or 31. December, 2006. One hundred and twenty (40%) patients died of vulvar cancer. The median follow-up time for patients still alive was 131 months (range 11 to 346 months). All tumors were staged based on the International Federation of Gynecology and the Obstetrics (FIGO) classification [16]. Approval of the study has been given by The Regional Committee for Medical Research Ethics South of Norway (S-06012), The Social- and Health Directorate (04/2639 and 06/1478) and The Data Inspectorate (04/01043).

Histological specimens were reviewed by J.M.N, one of the co-author, who was concealed from all clinical information. Classification was performed according to World Health Organization recommendations [17]. Two hundred and eighty-two (94%) tumors were keratinizing/nonkeratinizing, 14 (5%) were basaloid and 4 (1%) were veruccoid. Previously, we have examined the expression of 14-3-3σ and human papillomavirus (HPV) infection in primary vulvar carcinomas [18,19], which was compared with the expression of CDC25s from the present study. Ten samples of normal vulva form patients undergoing surgery for benign gynecological diseases were included as control.

### Cell Line

Two human vulvar squamous cell carcinoma cell lines, SW-954 (ATCC, Manassas, VA, USA) and CAL-39 (DSMZ, Germany), were cultured in RPMI 1640 medium (BioWhit-taker Europe, Verviers, Belgium) supplemented with 5% fetal bovine serum (FBS) (Biochrom KG, Berlin, Germany). For Western blot analysis and immunohistochemistry, monolayer cells were harvested by 0.01 M EDTA and thereafter washed in PBS.

### Immunohistochemical method

Four-μm sections made from formalin-fixed, paraffin-embedded tissues and cell lines were immunostained using the Advance™ HRP System (K4068, Dako Corporation, CA, USA). After deparaffinization, sections for CDC25A staining were microwaved in 10 mM Tris-1 mM EDTA, pH 9.0, sections for CDC25B and phospho-CDC25C (Ser 216) staining were microwaved in 1 mM EDTA, pH 8.0 and sections for CDC25C staining were microwaved in 10 mM citrate buffer, pH 6.0 to regain the epitopes blocked by formalin fixation. To block endogeneous peroxidase the sections were treated with 0.3% hydrogen peroxide (H_2_O_2_) for 5 min. Sections were incubated overnight at 4°C with monoclonal antibodies, including CDC25A (clone DCS-120+DCS-121, 1:500, 0.4 μg IgG_2a_/ml), CDC25B (clone 25B03, 1:150, 1.3 μg IgG_1_/ml), CDC25C (clone 25C07, 1:100, 2 μg IgG_1_/ml), all from NeoMarkers, CA, USA, and phospho-CDC25C (Ser 216) (clone 63F9, 1:500), from Cell Signaling, MA, USA. The specimens were then given a sequential 30 min incubation with Advance™ HRP link and Adance HRP enzyme, followed by treatment with 3'3-diaminobenzidine tetrahydrochloride (DAB) for 10 min, counterstained with hematoxylin, dehydrated and mounted in Diatex.

Sections from tonsil with known CDC25A, CDC25B and phospho-CDC25C (Ser 216) expression and from breast carcinoma with known CDC25C expression were used as positive control. Negative control included i) substitution of the monoclonal antibody with mouse myceloma protein of the same subclass and concentration as the monoclonal antibody, ii) incubation of sections with phospho-CDC25C (Ser 216) absorbed with phospho-CDC25C (Ser 216) peptide (Cell Signaling, MA, USA) as recommended by the supplier.

Semiquantitative classes were used to describe the intensity (absent, 0; weak, 1; moderate, 2; strong, 3) and extent of staining (percent of positive tumor cells: absent, 0; < 10%, 1; 10-50%, 2; > 50%, 3). By multiplying intensity score with extent score, product scores for both cytoplasm staining and nucleus staining were produced which ranging from 0 to 9. By taking product scores from cytoplasm and nucleus into account at the same time, a composed score was given for each section. Based on staining pattern observed in normal vulvar epithelium, cutoff values in cytoplasm and/or nucleus were set. High CDC25A and CDC25B immunostaining in the nucleus was classified with a score > 6, and low with a score ≤ 6, whereas, high CDC25C and phospho-CDC25C (Ser 216) immunostaining in cytoplasm was classified with a score > 3 and low with a score ≤ 3. In addition, high phospho-CDC25C (Ser 216) immunostaining in nucleus was classified with a score > 0 and low with a score 0. Examination of immunostaining was performed by two independent observers (Z.W. and R.H.) with no knowledge of patient outcome. All discordant scores were reviewed until final agreement was obtained.

### Protein extraction

Protein extraction was performed as described previously [20]. Cells were lysed in ice-cold NP-40 lysis buffer [1% NP-40, 10% glycerol, 20 mM Tris-HCl, pH 7.5, 137 mM NaCl, 100 mM sodium vanadate, 1 mM phenylmethylsulfonyl fluoride (PMSF) and 0.02 mg/ml each of aprotinin, leupeptin, pepstatin, and 10 μl/ml phosphatase inhibitor cocktail I (Sigma-Aldrich, St. Louis, MO)]. Lysates were sonicated and clarified by centrifugation. Protein quantitation was done by Bradford analysis. Twenty-five μg protein/lane was resolved by 12% SDS polyacrylamide gel electrophoresis (PAGE) and then Transferred to PVDF membranes.

### Western blot analysis

The PVDF membranes with protein extract from cell lines or 1 μg CDC25A, CDC25B and CDC25C antigens (Upstate, NY, USA) were blocked with 5% nonfat dry milk in tris-buffered saline-Tween (TBST) and subsequently hybridized with antibodies against CDC25s [CDC25A, 1:200; CDC25B, 1:200, (Santa Cruz Biotechnology, CA, USA); CDC25C, 1:200 and phospho-CDC25C (Ser 216), 1:500] overnight at 4°C, respectively. Membranes were then washed in TBST for 3 times, with 10 mins each, and further hybridized with corresponding anti-mouse or anti-rabbit IgG conjugated with Horseradish Peroxidase (HRP Labelled, 1:5000, 0.2 μg IgG_1_/ml dilution) for 1 hour at room temperature. After 3 times rince in TBST for 10 mins each, membranes were finally treated by Western Lightning Enhanced Chemiluminescence (ECL) reagent (Perkin Elmer, MA, USA).

### Nuclear and cytoplasmic extraction

The NE-REP nuclear and cytoplasmic Extraction Reagents (Pierce Biotechnology, Rockford, USA) was used to separate cytoplasm and nuclear proteins. Western blot analysis on each fraction was performed as described above. To confirm the pure separation of nuclear and cytoplasmic fractions, Lamin B, a nuclear protein exclusive recognizing antibody (Pierce Biotechnology), and Tubulin, a cytoplasmic protein exclusive recognizing antibody (Oncogene, San Diego, USA), were used, respectively.

### Protein dephosphorylation with CIAP

Proteins extracted from CAL-39 were dephosphorylated by Calf intestinal alkaline phosphatase (CIAP) (Promega, Madison, WI, USA). Proteins were treated by CIAP in a 50 μl reaction volume (protein 5 μg, CIAP 20 units, 10 × buffer 5 μl and H_2_O 42.5 μl). Following treatment for 30 min at 37°C, the reaction mixture was added an extra 20 units of CIAP and left for an additional 30 min at 37°C. Untreated and CIAP treated proteins were separated by SDS-PAGE and hybridized with CDC25C, phospho-CDC25C (Ser 216), CDC25A and CDC25B antibodies, respectively.

### Antigen phosphorylation with CHK1

One μg antigen CDC25B was phosphorylated by CHK1 (Millipore, Billerica, MA, USA) in a 50 μl reaction volume (antigen CDC25B 1 μg, CHK1 20 ng/μl, ATP 200 μM/μl, 10 × kinase buffer 5 μl and H_2_O 39 μl) for 30 min at room temperature. Untreated and CHK1 treated antigens were separated by SDS-PAGE, blotted onto PVDF membranes and hybridized with CDC25B and phospho-CDC25C (Ser 216) antibodies, respectively.

### Statistical analyses

Pearson's chi-square (χ^2^) test was performed in order to evaluate associations between CDC25 protein expression and clinicopathologic variables. Kaplan and Meier estimate and the log-rank test were used to evaluate and compare survival data. Disease-specific survival was calculated from the date of diagnosis to vulvar cancer related death. A Cox proportional hazards regression model was used for both univariate and multivariate evaluation of survival rates. In the multivariate analysis, a backward stepwise regression was performed with a *p *= 0.05 as the inclusion criterion for variables in the univariate analysis. All calculations were processed using SPSS 15.0 statistical software package (SPSS, Chicago, IL) and statistical significance was considered as *p *≤ 0.05.

## Results

### Specificity of antibodies

**Figure 1:**
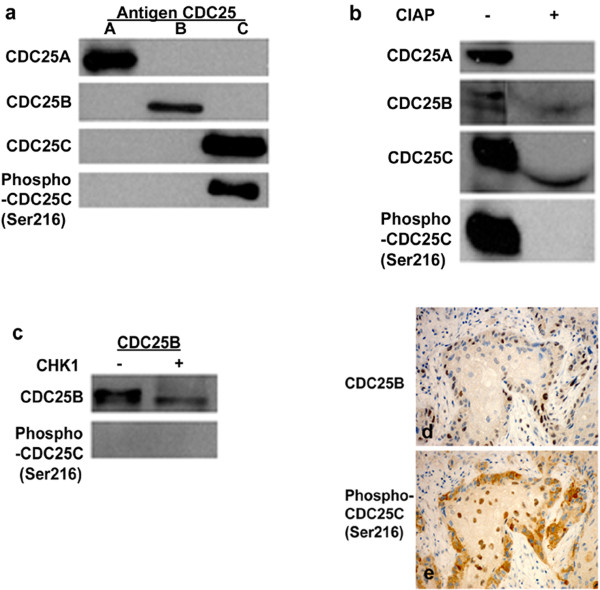
**Western blot analysis demonstrating specificty of CDC25s antibodies**. (a) One μg of CDC25A (lane 1), CDC25B (lane 2) and CDC25C (lane 3) antigens were immunoblotted with CDC25A, CDC25B, CDC25C and phospho-CDC25C (Ser216) antibodies, respectively. (b) Untreated (lane 1) and CIAP treated (lane 2) protein extracts from the vulvar cancer cell line CAL-39 were immunoblotted with CDC25A, CDC25B, CDC25C and phospho-CDC25C (Ser216) antibodies, respectively. (c) One μg of CDC25B antigen untreated (lane 1) and treated (lane 2) with CHK1 immunoblotted with CDC25B and phospho-CDC25C (Ser216) antibodies, respectively. Immunohistochemical staining of CDC25B (d) and phospho-CDC25C (Ser216) (e) on serial sections of vulvar carcinomas.

The specifity of CDC25 antibodies were tested by Western blot analysis using the antigenes CDC25A, CDC25B and CDC25C. However, since anti-CDC25B used for immunohistochemistry was not recommended by the supplier to use for Western blot analysis, an alternative anti-CDC25B was utilized for Western blot analysis. Our results showed that anti-CDC25A detected CDC25A, but not CDC25B or CDC25C. Anti-CDC25B (only for Western blot analysis) identified CDC25B, but not CDC25A or CDC25C. Anti-CDC25C and anti-phospho-CDC25C (Ser 216) immunoblotted with CDC25C, but not with CDC25A or CDC25B (Figure 1a). These results indicate that anti-CDC25A, anti-CDC25B, anti-CDC25C and anti-phospho-CDC25C (Ser 216) specifically detect their own respective antigen without any crossreaction with other CDC25s isoform.

Anti-CDC25C, as well as anti-CDC25B, detected proteins treated or un-treated with CIAP (Figure 1b), which indicate that anti-CDC25C and anti-CDC25B recognize their respective protein in phosphorylated as well as dephosphorylated form. However, proteins treated with CIAP failed to be identified by anti-CDC25A or anti-phospho-CDC25C (Ser 216) (Figure 1b), which indicate that both CDC25A and phospho-CDC25C (Ser 216) antibody exclusively detect phosphorylated CDC25A and CDC25C, respectively.

Anti-CDC25B detected CDC25B antigen treated or un-treated with CHK1, but neither of them was identified by anti-phospho-CDC25C (Ser 216) (Figure 1c). Furthermore, no coexpression of CDC25B and phospho-CDC25C (Ser216) was seen in serial sections of vulvar carcinomas (Figure 1d-e). These results indicate that the phospho-CDC25C (Ser 216) antibody does not cross-react with phosphorylated CDC25B.

### CDC25s protein expression

**Figure 2:**
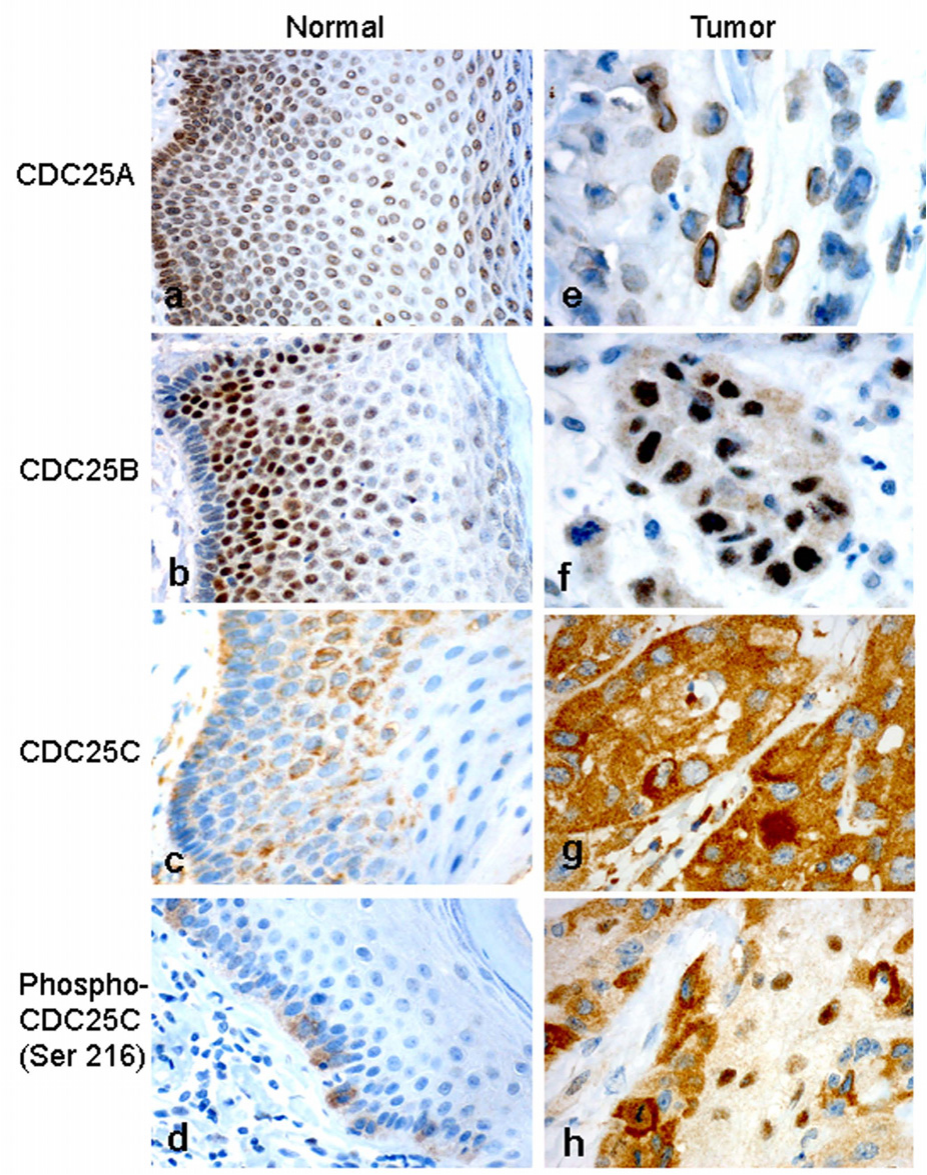
**Expression of CDC25 protein in vulvar squmous epithelium**. Immunohistochemical staining of CDC25A (a), CDC25B (b), CDC25C (c) and phospho-CDC25C (Ser216) (d) in normal vulvar epithelium. CDC25A (e), CDC25B (f), CDC25C (g) and phospho-CDC25C (Ser216) (h) staining in vulvar carcinomas.

In normal vulvar squamous epithelium, nuclear membrane staining for CDC25A was identified in basal, parabasal, middle and top layers (10/10 cases with score 9), whereas nuclear staining for CDC25B was seen in basal, parabasal and middle layers (10/10 cases with score 6) (Figure 2a-b). Cytoplasmic staining for CDC25C was observed in basal, parabasal and middle layers (3/10 cases with score 3 and 7/10 cases with score 6), whereas phospho-CDC25C (Ser 216) was limited to the basal layer (7/10 cases negative and 3/10 cases with score 3) (Figure 2c-d). None of the normal cases showed nuclear staining for CDC25C and phospho-CDC25C (Ser 216).

The immunostaining results in vulvar carcinomas are summarized in Table 1. High CDC25A and CDC25B immunostaining (score > 6) in the nucleus was observed in 152/300 (51%) and 47/300 (16%) cases, respectively (Figure 2e-f). CDC25A expression was observed in the nuclear membrane. High CDC25C expression (score > 3) in the cytoplasm was seen in 188/300 (63%) cases (Figure 2g). CDC25C immunostaining in the nucleus was not observed in any cases. High phospho-CDC25C (Ser216) expression in cytoplasm (score > 3) and nucleus (score > 0) were detected in 151/300 (50%) and 211/300 (70%) cases, respectively. Taking both the cytoplasmic and nuclear immunostaining into account, we found that high phospho-CDC25C (Ser216) immunostaining (score > 3) appeared in 232/300 (77%) cases (Figure 2h).

**Table 1:** Immunostaining results for CDC25s

Score	CDC25A	CDC25B	CDC25C	Phospho-CDC25C (Ser216)		
				
	Nucleus	Nucleus	Cytoplasm	Cytoplasm	Nucleus	Cytoplasm and nucleus
						
	n (%)	n (%)	n (%)	n (%)	n (%)	n (%)
0	3 (1.0)	2 (0.7)	13 (4.3)	54 (18.0)	89 (29.7)	18 (6.0)
2	0 (0)	6 (2.0)	5 (1.7)	15 (5.0)	7 (2.3)	8 (2.7)
3	35 (11.7)	36 (12.0)	94 (31.3)	80 (26.7)	46 (15.3)	42 (14.0)
4	1 (0.3)	6 (2.0)	17 (5.7)	17 (5.7)	10 (3.3)	13 (4.3)
6	109 (36.3)	203 (67.7)	135 (45.0)	125 (41.7)	112 (37.3)	143 (47.7)
9	152 (50.7)	47 (15.7)	36 (12.0)	9 (3.0)	36 (12.0)	76 (25.3)
Total	300 (100.0)	300 (100.0)	300 (100.0)	300 (100.0)	300 (100.0)	300 (100.0)

**Figure 3:**
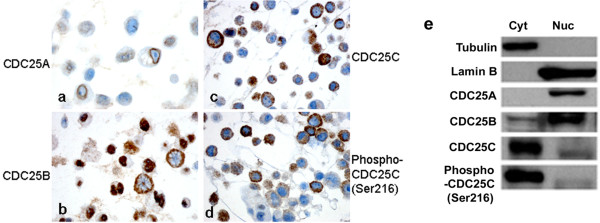
**Expression of CDC25 protein in vulvar cancer cell line**. Immunohistochemical staining of CDC25A (a), CDC25B (b), CDC25C (c) and phospho-CDC25C (Ser216) (d) in CAL-39 cell line. Western blot analysis of CDC25s cellular localization in CAL-39 cells (e). Cell fractions of cytoplasm (lane 1) and nucleus (lane 2) were immunoblotted with Tubulin and Lamin B antibodies to verify the purity of subcellular fraction extraction, then with CDC25A, CDC25B, CDC25C and phospho-CDC25C (Ser216) antibodies, respectively. Cyt: Cytoplasmic fraction; Nuc: Nuclear fraction.

In the vulvar carcinoma cell lines SW-954 and CAL-39, immunohistochemistry identified CDC25A (score = 3), CDC25B (score = 9), CDC25C (score = 3) and phospho-CDC25C (Ser216) (score = 3) in the nucleus, whereas, CDC25B (score = 3), CDC25C (SW-954, score = 6 and CAL-39, score = 9), and phospho-CDC25C (Ser216) (score = 9) were observed in the cytoplasm (Figure 3a-d). Similar results were detected using Western blot analysis (Figure 3e). CDC25A was seen only in the nuclear fraction. CDC25B was weakly expressed in the cytoplasmic and strongly in the nuclear fraction. In contrast, CDC25C and phospho-CDC25C (Ser 216) were strongly expressed in the cytoplasmic fraction, but weakly in the nuclear fraction.

### Immunostaining results of CDC25s in relation to clinicopathological parameters

High expression of CDC25A in the nucleus was significantly correlated to low expression of 14-3-3σ protein in cytoplasm, nucleus and cytoplasm/nucleus (*p *= 0.03, *p *= 0.01 and *p *= 0.01, respectively) and to low expression of phospho-CDC25C (Ser216) in nucleus and cytoplasm/nucleus (*p *= 0.002 and *p *= 0.04, respectively). High expression of CDC25B in the nucleus was significantly correlated with poor differentiation (*p *= 0.004), vessel infiltration (*p *= 0.03), high expression of phospho-CDC25C (Ser216) in cytoplasm and cytoplasm/nucleus (*p *= 0.01, and *p *= 0.04 respectively) and HPV infected cases (*p *= 0.03). The levels of CDC25C and phospho-CDC25C (Ser216) immunostaining in relation to clinicopathological parameters are shown in Table 2. High expression of CDC25C in cytoplasm was significantly correlated with high FIGO substage (*p *= 0.004), presence of lymph node metastases (*p *= 0.04), large tumor diameter (*p *= 0.03), poor differentiation (*p *= 0.03), high expression of 14-3-3σ protein in cytoplasm and cytoplasm/nucleus (both *p *= 0.001) and high expression of phospho-CDC25C (Ser216) in cytoplasm, nucleus and cytoplasm/nucleus (all *p *< 0.001). High expression of phospho-CDC25C (Ser216) in cytoplasm, nucleus and cytoplasm/nucleus were significantly correlated with high FIGO substage (*p *= 0.05, *p *= 0.01 and *p *= 0.005, respectively), large tumor diameter (*p *< 0.001, *p *= 0.009 and *p *< 0.001, respectively), deep invasion (*p *= 0.01, *p *= 0.01 and *p *< 0.001, respectively), high 14-3-3σ protein levels in cytoplasm (*p *= 0.007, *p *< 0.001 and *p *< 0.001, respectively) and high 14-3-3σ protein expression in cytoplasm/nucleus (*p *= 0.005, *p *< 0.001 and *p *< 0.001, respectively). Factors showing significant correlation with high expression of phospho-CDC25C (Ser216) in cytoplasm and cytoplasm/nucleus were poor differentiation (*p *< 0.001 and *p *= 0.01, respectively). High nuclear expression of 14-3-3σ was significantly correlated with high expression of phospho-CDC25C (Ser216) in nucleus (*p *< 0.001) and cytoplasm/nucleus (*p *= 0.002). Cases negative for HPV was significantly correlation with high expression of phospho-CDC25C (Ser216) in nucleus (*p *< 0.001).

**Table 2:** CDC25C and phospho-CDC25C (Ser216) immunostaining in relation to clinicopathological variables

Variables	Total	CDC25C			Phospho-CDC25C (Ser216)					
			
		Cytoplasm			Cytoplasm			Nucleus		
				
	n	Low	High (%)	*p*^1^	Low	High (%)	*p*^1^	Low	High (%)	*p*^1^
Age				0.91			0.91			0.45
25-69	119	46	73 (61)		61	58 (49)		40	79 (66)	
70-84	147	53	94 (64)		71	76 (52)		39	108 (74)	
85+	34	13	21 (62)		17	17 (50)		10	24 (71)	
FIGO				0.004			0.05			0.01
Ia	11	5	6 (55)		7	4 (36)		6	5 (46)	
Ib	35	23	12 (34)		24	11 (31)		17	18 (51)	
II	110	38	72 (66)		55	55 (50)		25	85 (77)	
III	121	38	83 (69)		55	66 (55)		32	89 (74)	
IV	19	6	13 (68)		6	13 (68)		6	13 (68)	
Not available	4									
Lymph node metastases				0.04			0.14			0.54
None	136	58	78 (57)		78	58 (43)		40	96 (71)	
Unilateral	76	21	55 (72)		33	43 (57)		19	57 (75)	
Bilateral	34	9	25 (74)		16	18 (53)		7	27 (79)	
Not available	54									
Tumour diameter (cm)				0.03			< 0.001			0.009
0.3-2.5	90	41	49 (54)		60	30 (33)		35	55 (61)	
2.6-4.0	94	34	60 (64)		46	48 (51)		21	73 (78)	
4.1-20.0	100	27	73 (73)		35	65 (65)		21	79 (79)	
Not available	16									
Tumor differentiation				0.03			< 0.001			0.30
Well	74	37	37 (50)		51	23 (31)		18	56 (76)	
Moderate	154	53	101 (66)		74	80 (52)		45	109 (71)	
Poor	72	22	50 (69)		24	48 (67)		26	46 (64)	
Depth of invasion (mm)				0.08			0.01			0.01
0.0-4.0	79	34	45 (57)		50	29 (37)		32	47 (60)	
4.1-8.0	98	27	71 (72)		48	50 (51)		21	77 (79)	
8.1-40.0	112	44	68 (61)		46	66 (59)		27	85 (76)	
Not available	11									
Infiltration of vessel				0.32			0.26			0.54
No	232	91	141 (61)		119	113 (49)		71	161 (69)	
Yes	65	21	44 (68)		28	37 (57)		17	48 (74)	
Not available	3									
HPV^2^				0.29			0.09			< 0.001
Low (-)	167	57	110 (66)		99	68 (41)		32	135 (81)	
High (+)	43	19	24 (56)		19	24 (56)		21	22 (51)	
Not available	90									
14-3-3σ cytoplasm^2^				0.001			0.007			< 0.001
Low (< 6)	83	43	40 (48)		52	31 (37)		39	44 (53)	
High (≥ 6)	217	69	148 (68)		97	120 (55)		50	167 (77)	
14-3-3σ nucleus^2^				0.40			0.48			< 0.001
Low (< 6)	123	42	81 (66)		58	65 (53)		51	72 (59)	
High (≥ 6)	177	70	107 (61)		91	86 (49)		38	139 (79)	
14-3-3σ cytoplasm/nucleus^2^				0.001			0.005			< 0.001
Low (< 6)	75	40	35 (47)		48	27 (36)		36	39 (52)	
High (≥ 6)	225	72	153 (68)		101	124 (55)		53	172 (76)	
CDC25A				0.48			0.49			0.002
Low	148	52	96 (65)		77	71 (48)		31	117 (79)	
High	152	60	92 (61)		72	80 (53)		58	94 (62)	
CDC25B				0.87			0.01			0.13
Low	253	95	158 (63)		134	119 (47)		74	179 (71)	
High	47	17	30 (64)		15	32 (68)		15	32 (68)	
CDC25C				-			< 0.001			< 0.001
Low	112	-	- -		76	36 (32)		47	65 (58)	
High	188	-	- -		73	115 (61)		42	146 (78)	

^1 ^Pearson chi-square

^2 ^In previous reports, 14-3-3σ and HPV have been studied [18,19].

**Figure 4:**
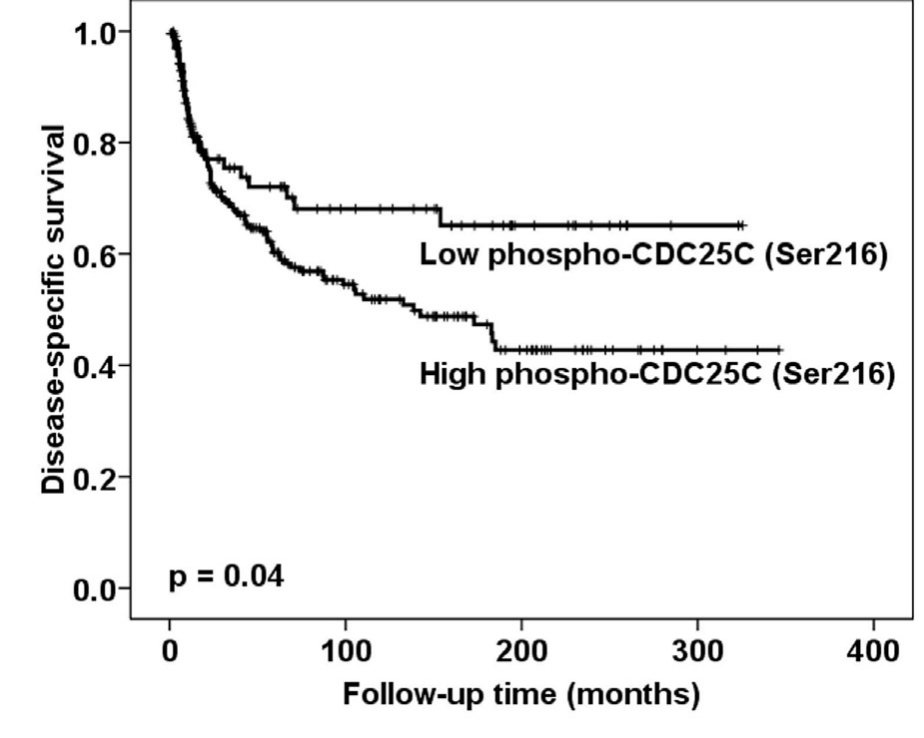
**Survival curves using the Kaplan-Meier method**. Kaplan-Meier curve of disease-specific survival in relation to phospho-CDC25C (Ser216) protein expression levels in cytoplasm/nucleus (*p *= 0.04) for the entire series (n = 300).

In the univariate analysis only high expression of phospho-CDC25C (Ser216) in cytoplasm/nucleus was associated with poor disease-specific survival (*p *= 0.04) (Figure 4). However, in multivariate analysis, when phospho-CDC25C (Ser216) cytoplasm/nucleus expression was added to the variables lymph node metastases, tumor diameter, infiltration of vessel, age and depth of invasion, only lymph node metastases, tumor diameter, vessel infiltration and age retained independent prognostic significance (Table 3).

**Table 3:** Relative risk (RR) of dying from vulvar cancer

Variables	Univariate analysis			Multivariate analysis		
		
	RR	95% CI^a^	*p*	RR	95% CI^a^	*p*
Lymph node metastases	2.49	1.92-3.23	< 0.001	2.18	1.64-2.90	< 0.001
Tumour diameter	1.78	1.40-2.25	< 0.001	1.47	1.10-1.95	0.009
Infiltration of vessel	2.42	1.64-3.57	< 0.001	1.76	1.10-2.82	0.02
Age	1.60	1.22-2.10	0.001	1.46	1.03-2.08	0.03
Phospho-CDC25C (Ser216)^b^	1.64	1.02-2.64	0.04	-	-	-

^a ^95% confidence interval

^b ^Cytoplasma/nucleus: low ≤ 3 and high > 3

## Discussion

Overexpression of CDC25A has been reported in breast [12], esophageal [14], hepatocellular [21], colorectal [9] and ovarian carcinomas [13]. Therefore, abnormal expression of CDC25A was implied as one of the potential oncogenic factors leading to dysregulation of cell cycle control and consequently oncogenic transformation. However, in our study overexpression of CDC25A was found not only in 51% of the vulvar carcinomas, but also in normal vulvar squamous epithelium, indicating that high expression of CDC25A is not important in the pathogenesis of vulvar carcinomas. In agreement with studies on thyroid [22] and colorectal carcinomas [9], we found that disease-free survival was not significantly associated with high expression of CDC25A, although such an association was reported in breast [12], oesophageal [14] and hepatocellular carcinomas [21]. These results indicate that the role of CDC25A is cancer specific.

In our study, 16% of the vulvar carcinomas have higher expression of CDC25B than normal vulvar squamous epithelium. Previously, a wide range of CDC25B overexpression (20-79%) has been reported in many other cancer types [5]. This discrepancy may be due to the various tumor types studied. Overexpression of CDC25B in 16% of our cases suggests that this protein may contribute to tumorigenesis in a minority of vulvar carcinomas. We failed to find an association with high CDC25B expression and disease-free survival, which was also reported in studies on prostate [23], gastric [24], hepatocellular [21], breast [12] and endometrial carcinomas. In contrast, a positive correlation was found in colorectal [9] and ovarian carcinomas [13]. These results suggest that controversy still rests on CDC25B as a universal tumor marker and that its prognostic value on gynaecologic carcinomas seems limited.

In previous studies, overexpression of CDC25C has been reported in a limited number of carcinomas. Therefore, CDC25C was regarded as a less oncogenic factor than CDC25A and CDC25B [5]. However, in the present study high expression of CDC25C was observed in 63% of vulvar carcinomas, a finding in line with studies on prostate [15], colorectal [25] and endometrium carcinomas [10]. The fact that overexpression of CDC25C was associated with advanced FIGO stage, presence of lymph node metastases, large tumor diameter and poor differentiation indicate that high level of CDC25C is an event occuring late in the tumor development. Our study failed to show a positive correlation between overexpression of CDC25C and disease-specific survival, which was similar to the results reported in esophageal [26], pancreatic [27], gastric [24] and ovarian carcinomas [13].

Compared to the low level of cytoplasmic phospho-CDC25C (Ser 216) protein expression in basal layers of normal vulvar squamous epithelium, high phospho-CDC25C (Ser 216) protein expression was found in the cytoplasm of 50% and in the nucleus of 70% of vulvar carcinomas. High expression of cytoplamic phospho-CDC25C (Ser 216) was correlated with high level of cytoplasmic 14-3-3σ. This strengthens the theory that phospho-CDC25C (Ser 216) protein sequestrated in the cytoplasm due to binding of 14-3-3 lose its access to nuclear CDK1/cyclin B complex, thus inhibiting mitotic entry [5]. Unexpectly, high expression of nuclear phospho-CDC25C (Ser 216) was correlated with high expression of nuclear 14-3-3σ protein. One hypothesis is that phospho-CDC25C (Ser 216) in the nucleus is unable to bind 14-3-3σ [28]. Phospho-CDC25C (Ser 216) will then still stay in the nucleus and activate CDK1/cyclin B complex, thus triggering G2/M transition. Therefore, high nuclear expressions of phospho-CDC25C (Ser 216) in the majority of cases indicate that phospho-CDC25C (Ser 216) may be important in the carcinogenesis of vulvar carcinomas and would be a potential target for cancer therapy.

According to the analyses between phospho-CDC25C (Ser 216) and clinical parameters, high expression of phospho-CDC25C (Ser 216) in cytoplasm/nucleus was significantly correlated with advanced FIGO stage, large tumor diameter and deep invasion as well as poor disease-specific survival. However, such an association between phospho-CDC25C (Ser 216) expression in cytoplasm/nucleus and disease-specific survival was annulled in multivariate analysis. The phospho-CDC25C (Ser 216) has to our knowledge not been previously investigated in any human cancer. Therefore, further studies are needed to clarify the role of phospho-CDC25C (Ser 216) as a prognostic marker.

In the present study, overexpression of CDC25A, CDC25B and CDC25C isoforms was not significant associated with each other, suggesting that overexpression of multiple isoforms in vulvar carcinomas occur through independent pathways [5]. However, high phospho-CDC25C (Ser 216) expression was correlated with low expression of CDC25A and high expression of CDC25B, suggesting that the three can collaborate in the tumorigenesis of a subset of vulvar carcinomas. Firstly, low expression of CDC25A in 49% of vulvar, compared to its high expression in normal tissues, might account for DNA damage-induced G2 arrest which is accompanied by proteasome-dependent destruction of CDC25A [7,29]. Secondly, CDC25C phosphorylated at Ser 216 in response to DNA damage [7] stay in the nucleus, instead of being sequestrated in cytoplasm, and might still be able to phosphorylate its substrate CDK1/cyclin B complex, resulting in an un-thorough G2 arrest. This un-thorough G2 arrest might be enhanced by overexpression of CDC25B which, other than A and C, was essential for mitotic entry as cells recover from a DNA-induced checkpoint arrest [7,30-33].

Our result showed that infection of HPV correlated with high expression of CDC25B and nuclear phospho-CDC25C (Ser216). This result is in agreement with previous studies of CDC25B, where CDC25B mRNA was highly elevated in fibroblasts after being transformed by SV-40 or by E6 or E7 papilloma virus transforming proteins [5,34]. Little is known about CDC25C post-transcriptional changes due to virally induced cellular transformation. However, we found an increased CDC25C protein expression in presence of HPV infection. In our study we could not demonstrate an association between CDC25A protein and HPV infection, although an elevated mRNA level and enzyme activity of CDC25A was found in quiescent human fibroblasts infected with the EIA adenovirus protein [35]. These findings suggest that CDC25s promoters may be specifically targeted by viruses during the cell transformation process [5] and that CDC25B and CDC25C may be subjected to HPV regulation.

Human CDC25 proteins consist of two domains: the N-terminal regulatory domain where the three isoforms share 20-25% identity and the C-terminal catalytic domain sharing approximately 60% identity [7]. Due to the similarity in the structure, antibodies to CDC25A, CDC25B and CDC25C have a high potential to not only identify their specific antigen, but also the other two isoforms. We found that the four CDC25 antibodies used in the present study detected their own respective antigen without any cross-reaction with the other CDC25s, strengthening the trustiness of our results. In contrast, previous papers have used CDC25s antibodies for immunohistochemistry without knowing the specificity of their antibodies [8,10,14,22,23,27,36,37], which may partly explain the conflicting results regarding cellular localization of CDC25A, CDC25B and CDC25C [6].

Interestingly, a different immunostaining pattern was seen between the two CDC25C antibodies in vulvar carcinomas. By using anti-CDC25C, which recognized both phosphorylated and dephosphorylated forms, immunostaining was detected only in cytoplasm, whereas anti-phospho-CDC25C (Ser 216), which only recognizes phosphorylated form, immunostained both in cytoplasm and nucleus. Previously, it has been reported that six amino acids are homologous in the phospho-CDC25C (Ser 216) and phospho-CDC25B (Ser323) domain [38]. Therefore, we were led to believe that the nuclear immunostaining observed by using anti-phospho-CDC25C (Ser 216) was false due to cross-reaction with phospho-CDC25B (Ser323). However, this was not the case, since we excluded the possibility that anti-phospho-CDC25C (Ser 216) cross-react with phosphorylated CDC25B by the kinase test and immunostaining. On serial sections, no coexpression was observed between phospho-CDC25C (Ser 216) and CDC25B, both phosphorylated and dephosphorylated forms. Therefore, we speculated that in vulvar carcinomas the epitope in the phospho-CDC25C (Ser 216) domain supposed to be recognized by anti-CDC25C (phosphorylated and dephosphorylated) may be masked for some unknown reasons resulting in lost nuclear staining.

## Conclusions

Our results suggest that CDC25C and phospho-CDC25C (Ser216) play a crucial role and CDC25B a minor role in the development and/or progression of vulvar carcinomas. CDC25B, CDC25C and phospho-CDC25C (Ser216) expression were associated with malignant features and aggressive cancer phenotypes. However, the CDC25s isoforms were not independently correlated to prognosis.

## Competing interests

The authors declare that they have no competing interests.

## Authors' contributions

ZW participated in the design of the study, carried out the immunohistochemistry, immunoblotting, statistical and data analysis and draft the manuscript. CGT collected clinical data, participated in interpretation of data and helped to draft the manuscript. VAF participated in immunoblotting analysis and interpretation and revised the manuscript critically. ZS participated in the design of the study and revised the manuscript critically. JMN performed systematic pathologic review of vulvar carcinomas and revised the manuscript critically. RH participated in the design of the study, protein, statistical and data analysis and helped to draft the manuscript. All authors read and approved the final manuscript.

## Pre-publication history

The pre-publication history for this paper can be accessed here:

http://www.biomedcentral.com/1471-2407/10/233/prepub
